# Wild mushrooms showed analgesic and cytotoxic properties along with phytoconstituent's binding affinity to COX-1, COX-2 and cytochrome P450 2C9

**DOI:** 10.1016/j.heliyon.2021.e07997

**Published:** 2021-09-14

**Authors:** S.M. Moazzem Hossen, Mohammad Shahadat Hossain, Sumaiya Akbar, Umme Tahmida, Jannatul Mawa, Nazim Uddin Emon

**Affiliations:** a Department of Pharmacy, Faculty of Biological Science, University of Chittagong, Chittagong 4331, Bangladesh; b Department of Pharmacy, Faculty of Science and Engineering, International Islamic University Chittagong, Chittagong 4318, Bangladesh

**Keywords:** Wild mushrooms, Analgesic, Cytotoxic, Receptors, *In silico*, Molecular docking

## Abstract

This study was designed to evaluate the cytotoxic and analgesic potential of methanol extracts of five wild mushrooms available in the University of Chittagong, Bangladesh. The acetic acid-induced writhing method was used for the analgesic activity, while cytotoxicity was tested using brine shrimp lethality bioassay. *In silico* molecular docking and ADME/T study have been employed by using Schrodinger v11.1, BIOVIA Discovery Studio 2020 and online tool (AdmeSAR) respectively. The methanol extracts of *Daldinia concentrica*, *Trametes lactinea*, *Fomitopsis cajanderi* and *Daedaleopsis confragosa* exhibited a significant (*p* < 0.001) decrease in the number of writhing when compared to the control group. Except for *Lentinus squarrosulus* at 200 mg/kg body weight, the remaining mushroom extracts showed equal to or above 50 % inhibition of writhing. *Daldinia concentrica* showed the lowest LC_50_ values with 0.63 μg/mL, while *Daedaleopsis confragosa* showed the highest LC_50_ values of 2.33 μg/mL, indicating decisive cytotoxic action all mushrooms extracts. Considering the secondary metabolites, daldinan A and fomlactone A were found the most promising myco-compounds in analgesic and cytotoxic molecular docking studies. Besides, all the selected metabolites meet the rule of Lipinski's drug-likeliness. These results concluded that each mushroom extracts except *Lentinus squarrosulus* possess a potential analgesic. All the mushroom extracts embrace a promising cytotoxic activity that may guide the progress of a new drug.

## 1 Introduction

From ancient civilization to modern times, mushrooms have been used as food for their enormous nutritional values (Bhunia et al., 2010; Rathore et al., 2017). Furthermore, mushrooms possess notable medicinal effects as the fungi (Gargano et al., 2017). Nowadays, they are widely utilized in the nutraceuticals, pharmaceuticals, and cosmeceuticals sector Rathore et al. (2017). Several studies have reported that mushrooms contain bioactive agents which are responsible for showing different medicinal effects like anti-diabetic (Wu and Xu, 2015), antibacterial (Beltran-Garcia et al., 1997), antiviral (Teplyakova and Kosogova, 2016), antioxidant (Liu et al., 2014), antitumor (Meng et al., 2016), immunomodulatory and hepatoprotective (Soares et al., 2013) effects.

The popularity of mushrooms for pain management has been increasing because of their tremendous analgesic effects (Sajon et al., 2018). According to the researchers, several mushrooms can inhibit cancer progression by exerting their cytotoxic actions on cancer cells (Chatterjee et al., 2017). *Lentinus squarrosulus (*Mont.) Singer (Polyporaceae) usually develops on logs and dead leaves. The fungi comprise 22.82% of crude proteins, 2.76% of moistures, 6.29% of crude fats, and 7.52% of ashes (Nwanze et al., 2006). Polysaccharides of mushrooms are the most effective antitumor candidates in the therapeutic practice (Kishida et al., 1992). Many glucans and heteroglycans have already been extracted in the lab from certain edible mushrooms (Bhunia et al., 2010). Lentinan 10 (b-glucan), extracted from the *Lentinula edodes* demonstrates conspicuous antitumor action (Hamuro, 1985) and is commercially available around the globe. *Trametes lactinea* (Berk.) Pat, (Polyporaceae) is a wood-rotting fungus which is a kind of polypore that grows in rotten wood. Previous research of *Trametes lactinea* (showed that this mushroom contains a biologically active constituent, namely Trametenolic acid B, which effectively suppressed the gastric cancer cells through H + -K + ATPase inhibitory activities (He et al., 2018; Zhang et al., 2013). *Daedaleopsis confragosa* (Polyporaceae) has an abundance of fatty acids and lipids. For instance, it contains glycolipids 13.3%, phospholipids 53.8%, neutral lipids 32.9%, and 20.1% total lipid (Dembitsky et al., 1993; Pemberton, 1994)*.* It contains various antioxidants that can scavenge free radicals and inhibit oxidative damage (Yan and Chang, 2012). *Fomitopsis cajanderi* (Fomitopsidaceae), a polypore with a pretty pink pore, has been founded in the conifer forests of North America (Kuo, 2010). *Daldinia concentrica* (Hypoxylaceae) is widely spread throughout the world, mostly in the temperate climate. On the dead, dying logs, the fruit bodies are individually or dispersed and easily identified. This fungus is used to cure pneumonia and other bacterial infections as conventional therapies in Yorubaland, Western Nigeria (Goswami et al., 2020).

Pain is a physical, cognitive, and unpleasant occurrence that is considered a global health problem (Paliwal et al., 2017). Mainly, disease or an injury launches the pain in the body (Mills et al., 2019). According to the IASP's revised definition, pain is an uncomfortable sensory and emotional sensation related to defined or possible tissue damage (Raja et al., 2020). Several pathways are involved in inducing pain, such as transduction, transmission, modulation, and perception. Pain is managed by targeting the pathways using analgesics (Van Rensburg and Reuter, 2019). Opioids and non-steroidal anti-inflammatory drugs (NSAIDs) are the most widely used analgesics of modern times. They can alleviate half of the pain in around 30 % of the patient (Rahaman et al., 2020). Opioids work by adhering to opioid receptors (Rosenblum et al., 2008). NSAIDs usually act by hindering the synthesis of prostaglandins through the inhibition of cyclooxygenase enzymes (Gunaydin and Bilge, 2018). NSAID's have been particularly useful in pain and inflammatory treatment, but these agents are well known to be associated with the gastrointestinal toxicity. The inhibitors of cyclo-oxygenase 2 can also decrease the risk of gastrointestinal events but they also claimed to have negative cardiovascular events (Stalnikowicz and Rachmilewitz, 1993). Serious complications caused by NSAIDs including hemorrhage, perforation and death arise collectively with a yearly estimated rate of roughly 2% for NSAID users (De Cosmo and Congedo, 2015). Again, opioids usually cause addiction, tolerance and physical dependence. Using opioids for a longer period of time results in dependency such as physical (e.g; such as muscle cramping, diarrhea, and anxiety) and psychological signs of depression when people quit using the opoids (Hijazi et al., 2017; Kosten and Baxter, 2019).

As a result, researchers have been investigating more natural sources to find alternatives to current pain medications (Shojaii et al., 2015). In addition, uncontrolled propagation of abnormal cells is known as cancer. Nowadays, it is one of the most serious health problems which cause death (Glanville et al., 2015). The use of cytotoxic drugs in cancer chemotherapy is frequent (Soumya et al., 2011). Synthetic anticancer drugs are expensive and they can cause toxicity and adverse side effects. For that, anticancer drugs obtained from natural sources are the most important alternatives (Ayaz et al., 2016). Forthermore, At the present time, the popularity of docking as a standard computational technique for the identification of new active compounds and their affinities to particular receptors has become ubiquitous (Rudra et al., 2020). Cyclooxygenase-1, cyclooxygenase-2 and cytochrome P450 2C9 are well recognized proteins those are associated with the pain, inflammation and tumour (Emon et al., 2021b; Williams et al., 2003a).

Owing to the tremendous pharmaceutical application of mushrooms, as per our best knowledge there is no record of analgesic and cytotoxic investigations regarding the selected wild mushrooms. However, in this research we sought to investigate the analgesic and cytotoxic activity of five wild mushrooms (*Lentinus squarrosulus, Daldinia concentrica, Trametes lactinea, Fomitopsis cajanderi* and *Daedaleopsis confragosa*) found in the University of Chittagong through in vivo and in silico approaches.

## 2 Materials and methods

### 2.1 Drugs and reagents

All the chemicals were of analytical grade purity. Methanol (Merck, Germany), acetic acid (Fluka Chemica, Switzerland), diclofenac sodium (Square Pharmaceuticals Ltd., Bangladesh), 0.9% NaCl (Beximco Pharma, Bangladesh), Tween 80 (Sigma-Aldrich) and vincristine sulfate (Beacon Pharmaceuticals Ltd., Bangladesh) were used in this study.

### 2.2 Collection and identification of the mushrooms

Five wild mushrooms, including *Lentinus squarrosulus, Daldinia concentrica, Trametes lactinea, Fomitopsis cajanderi* and *Daedaleopsis confragosa* were collected from different areas of the Chittagong University campus, Bangladesh. Mushroom Specialist Dr. Akhter Jahan Kakon, Mushroom Research Centre, Savar, Dhaka, identified and preserved the specimens of the mushrooms. The accession numbers of the mushroom specimens are as follows: *Lentinus squarrosulus* (2018/007/Fungi/CU/DP), *Daldinia concentrica* (2018/008/Fungi/CU/DP), *Trametes lactinea* (2018/009/Fungi/CU/DP), *Fomitopsis cajanderi* (2018/010/Fungi/CU/DP), *Daedaleopsis confragosa* (2018/011/Fungi/CU/DP).

### 2.3 Preparation of extract

Completely shade-dried mushrooms were milled to the powders. Subsequently, to the clean glass containers, we entered approximately 100 g of powder of each mushroom to soak in 500 mL of methanol solvent. After sealing the containers, they were reserved for one week following random shaking. The mixtures then experienced filtration by Whatman filter paper no 1. A rotary evaporator was to evaporate the filtrates. A gummy concentrated black color residue of methanol extracts found with a yield of *Lentinus squarrosulus* 13 g, *Daldinia concentrica* 17 g, *Trametes lactinea* 15 g, *Fomitopsis cajanderi* 14 g and *Daedaleopsis confragosa* 18 g, respectively. These extracts were kept in tightly closed glass containers and stored in the refrigerator for further use and the extracts were diluted to different concentrations by using 1% tween 80 as a vehicle.

### 2.4 Experimental animals

Four to five weeks adult Swiss-albino mice of either sex (male and female) (20–25 g weight) procured from the BCSIR laboratories, Chattogram to perform the *in vivo* studies. At least one week before commencing the experiment, the rodents were housed in clean cages of the Animal House of Department of Pharmacy of the University of Chittagong at room temperature (23 ± 2 °C) with around 12 h light-dark cycle acclimatize with the environment. Standard laboratory diets (5 g of pellets ration; ground wheat, ground corn, di-calcium phosphate, mono-calcium phosphate, choline chloride, zinc oxide, ferrous carbonate, casein, folic acid, vitamin B12), and water ad libitum were provided to the mice. Laboratory animal clinical trial approved by Departmental ethical review committee, Department of Pharmacy, University of Chittagong, Chittagong 4331, Bangladesh under the consent number: CUDP:17/03/2019:28.

### 2.5 Acute oral toxicity test

After administering single or multiple doses of substances, several adverse reactions may appear, which dictate acute toxicity. OECD guidelines (up and down method) (Rispin et al., 2002) were followed to determine the LD_50_ of the test specimens. After the oral administration of different concentrations of the test extracts (100, 200, 400, 1000, 2000 and 3000 mg/kg body weight) to the animals (5 mice for each dose) of both sex were observed to record any sign of toxicity or death for 1 h. Rodents were monitored for the following 5–6 h on each hour basis. After that, for the next two weeks, the mice were kept under close monitoring (Aziz et al., 2019; Emon et al., 2021b).

### 2.6 Analgesic activity

The acetic acid-induced writhing test was used to determine the analgesic activity of the extracts of mushrooms (Alam et al., 2020; Koster, 1959). Sixty mice (both sex) were divided into twelve groups, where each group consists of five mice. Negative control group, positive control group and test groups received vehicle (1% Tween-80 in saline, 10 mL/kg; body weight), diclofenac sodium (10 mg/kg; body weight) and two different doses (200 and 400 mg/kg; body weight) of the test extracts respectively. Forty minutes after the oral administration of the samples, 0.7% acetic acid was administered intraperitoneally to induce pain in mice. The cumulative quantities of writhing were calculated for the individual animal for 15 min, 5 min after administering acetic acid. The percentage of inhibition was determined using the following equation (Equation 1):

(1) $$\%\text{Inhibition}=\left(\frac{Wc-Wt}{Wc}\right)\times 100$$

where W_c_ = average number of writhing in the control group and W_t_ = average number of writhing in the test groups.

### 2.7 Cytotoxic activity

Brine shrimp nauplii lethality test was conducted to determine the cytotoxicity of mushroom extracts (Hasanat et al., 2019; McLaughlin, 1982). Trirty-eight grams of NaCl (3.8%) was dissolved in 1 L of distilled water to prepare the artificial seawater. A clear solution was obtained by filtering this artificial seawater. Hatched brine shrimps were collected from the Faculty of Biological Science, University of Chittagong were observed after two days of continuous aeration in artificial seawater. At first, the extracts were dissolved in seawater with DMSO (<0.01%) and then shifted to test tubes to achieve concentrations of 50, 100, 250, 500 and 700 μg/mL in 5 mL simulated seawater with ten shrimps in every test tube. Vincristine sulfate was operated as a positive control at deficient concentration (5, 1, 0.5, 0.25 μg/mL) as it is a strong cytotoxic alkaloid. DMSO in simulated seawater at the same attention as the samples was used in the case of control test tubes. A magnifying glass was used to count the number of shrimps alive after 24 h incubation at room temperature (23 ± 2 °C). To calculate the % of mortality following equation was used (Equation 2):

(2) $$\%\text{Mortality}=\frac{Nt-Na}{Nt}\times 100$$

Where N_t_ is the number of shrimps transferred (n = 10) and N_a_ is the number of shrimps alive after 24 h incubation period. After that, the median lethal concentration (LC_50_) of the samples was estimated.

### 2.8 *In silico* study

#### 2.8.1 Molecular docking: ligand preparation

**Figure 1:**
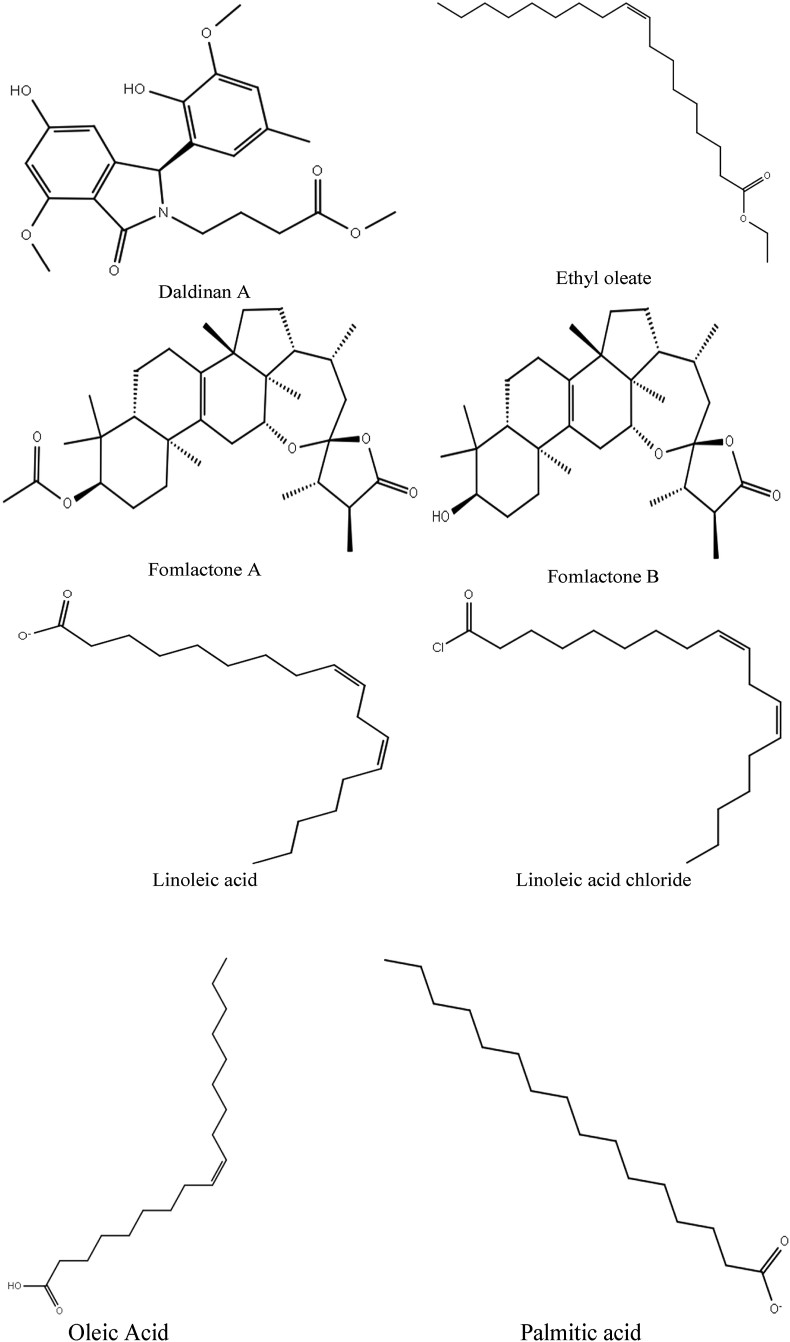
Structures of the phytocontituents from the selected wild mushrooms.

The structure of eight major isolated compounds namely ethyl oleate, palmitic acid from the ethanol extract of *L. squarrosulus* (Adeoye-Isijola et al., 2018), linoleic acid, linoleic acid chloride and oleic acid from the acetone extract of mycelia of *T. lactinea* (Yahaya and Don, 2012), daldinan A from the methanol extract of the fruiting body of *D. concentrica* (Kim et al., 2013; Lee et al., 2012), fomlactone A and fomlactone B from the petroleum ether and diethyl ether extract of fruit body of *Fomitopsis cajanderi* (He et al., 2006) (Figure 1) were retrieved from the PubChem database. The ligands structures were plotted two-dimensionally (2D) by utilizing the LigPrep in Maestro version 11.1 (Schrödinger suite, LLC New York, NY, USA) with the force field OPLS_2005 and pH 7.0 ± 2.0 for ionization state generation that used Epik 2.2.

#### 2.8.2 Molecular docking: protein preparation

2D structures of the protein used for the experiments include cyclooxygenase-1 (PDB ID: 2OYE) (Harman et al., 2007) and prostaglandin synthases 2 (PDB ID: 6COX) (Kurumbail et al., 1996) for the analgesic and crystal structure of human cytochrome P450 2C9 (PDB ID: 1OG5) (Williams et al., 2003b) for the cytotoxic evaluations which were collected from RCBS Protein Data Bank in PDB format. Thereupon, the structures were prepared and refined by applying Protein Preparation Wizard (Schrödinger-Maestro version 11.1). Analogously, charges and bond orders were assigned, hydrogens were attached to the heavy atoms, selenomethionines were altered to methionine, and waters were eliminated.

#### 2.8.3 Molecular docking: glide standard precision

Molecular docking studies were performed to elucidate the possible mechanism of the selected compounds against COX-1, COX-2 and crystal structure of human cytochrome P450 2C9 enzymes for analgesic and cytotoxic analysis. Docking experiments were carried out using Glide embedded in Maestro by standard precision scoring function as we previously described (Emon et al., 2020). In Glide, grids were generated by the default scaling factor of van der Waals (1.00) and workload control factor of 0.25, optimized for the force field OPLS 2005. For the receptor, the size of the cabinet was set to 14 Å × 14 Å × 14 Å. Molecular studies of docking were measured by recording each ligand's best-docked position with the lowest glide score. Finally the best binding interactions have been visualized in Discovery studio 2020.

#### 2.8.4 Pharmacokinetics and toxicity measurement

Here for determining the pharmacokinetic properties (ADME) of three major compounds, the online tool SwissADME (http://www.swissadme.ch/) was used. Lipinski's rule of five (Molecular weight not more than 500; H-bond donors ≤5; H-bond acceptors ≤10; Lipophilicity <5 and molar refractivity ranging from 40-130) were considered to evaluate favorable drug-like properties of all compounds (Lipinski et al., 1997). Moreover, the toxicological properties of all the compounds were determined by the web tool admetSAR (http://lmmd.ecust.edu.cn/admetsar2/).

## 3 Statistical analysis

The data are presented as mean ± standard error mean (SEM). The significance of analgesic activity of the extracts of mushrooms was determined by using the one-way analysis of variance (ANOVA) test, followed by Dunnet's t-test (2-sided) compared with the control. Values of *P* < 0.001 were considered significant. The data were analyzed using SPSS (Statistical Package for the Social Sciences) program (version 16.0 SPSS Inc., Chicago, IL, USA). The median lethal dose (LC_50_) values were calculated using GraphPad Prism software version 6.01 (GraphPad Software, San Diego, CA, USA).

## 4 Results

### 4.1 Acute oral toxicity test

No sign of toxicity was found at the test doses. Furthermore, none of the mice died during this toxicity test. Daily fluctuations in the intake of food and water of animals were controlled. So, the LD_50_ of the mushroom extracts was determined to be greater than 3000 mg/kg. This shows that the mushroom extracts were stable at a single bodyweight dose till 3000 mg/kg. Considering the acute oral toxicity test results and the safety of the doses, 200 and 400 mg/kg; b.w, p.o doses for the analgesic activity were chosen.

### 4.2 Analgesic activity

**Figure 2:**
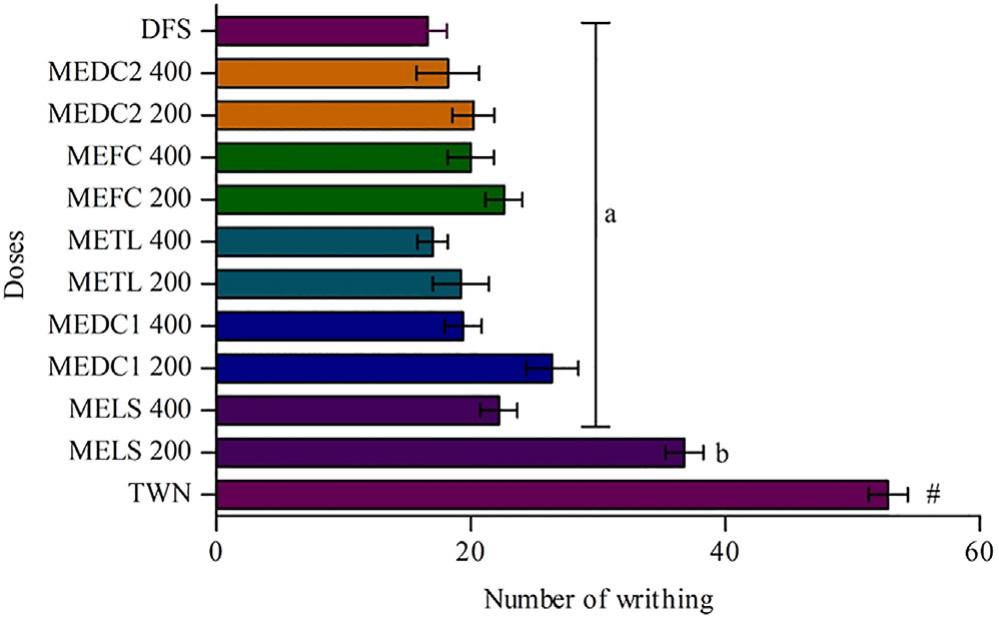
Values are expressed as mean ± SEM or percentage and statistically analyzed using ANOVA followed by Dunnett's t test, n = 5. ^b^*p* < 0.01, ^a^*p* < 0.001, when compared to control. TWN = Tween 80, MELS = Methanol Extract of *Lentinus squarrosulus*, MEDC1 = Methanol Extract of *Daldinia concentrica*, METL = Methanol Extract of *Trametes lactinea*, MEFC = Methanol Extract of *Fomitopsis cajanderi*, MEDC2 = Methanol Extract of *Daedaleopsis confragosa*, DFS = Diclofenac Sodium.

In this acetic acid-induced test, a significant (*p* < 0.001) dose-dependent decrease in the number of the writhing of mice found at both doses (200 mg/kg and 400 mg/kg body weight) of all mushrooms extracts when compared to the control group. All the mushroom extracts exhibited equal or above 50 % inhibition of writhing in acetic acid-induced mice except *L. squarrosulus* (30.30%) at 200 mg/kg body weight dose. The test samples showed a concentration-dependent analgesic activity by inhibiting the writhing numbers with the writhing numbers of 52.8 ± 1.56 and 16.6 ± 1.50 for the negative and positive controls, 36.8 ± 1.50 and 22.2 ± 1.46 for the MELS (200 and 400 mg/kg, b.w; p.o), 26.4 ± 2.04a and 19.4 ± 1.44 for the MEDC1 (200 and 400 mg/kg, b.w; p.o), 19.2 ± 2.20 and 17.0 ± 1.18 for the METL (200 and 400 mg/kg, b.w; p.o), 22.6 ± 1.44 and 20.0 ± 1.82 for the MEFC (200 and 400 mg/kg, b.w; p.o), 20.2 ± 1.66 and 18.2 ± 2.44 for the MEDC2 (200 and 400 mg/kg, b.w; p.o). The results of this experiment are summarized in Figure 2.

### 4.3 Cytotoxic activity

The cytotoxic effects of the test extracts are depicted in Table 1. *D. Concentrica* showed the lowest LC_50_ value of 0.63 μg/mL, while *D. confragosa* showed the highest LC_50_ value of 2.33 μg/mL. The LC_50_ value of the remaining mushrooms named *L. squarrosulus*, *T. lactinea* and *F. cajanderi* was 1.42 μg/mL, 1.27 μg/mL and 1.64 μg/mL, respectively. When the concentrations of the extracts increased, the mortality rate of the shrimp nauplii also increased.

**Table 1:** Effect of different mushrooms extracts on brine shrimp nauplii.

Treatment	Conc. (μg/mL)	% of mortality	LC_50_ (μg/mL)	Regression equation	R square
MELS	50	60	1.42	Y = 37.58∗X - 3.496	0.9770
	100	70			
	250	90			
	500	100			
	700	100			
MEDC^1^	50	70	0.63	Y = 23.50∗X + 35.28	0.8406
	100	90			
	250	90			
	500	100			
	700	100			
METL	50	60	1.27	Y = 33.99∗X + 6.872	0.9418
	100	80			
	250	90			
	500	100			
	700	100			
MEFC	50	50	1.64	Y = 43.71∗X - 21.76	0.9692
	100	70			
	250	80			
	500	100			
	700	100			
MEDC^2^	50	0	2.33	Y = 96.67∗X - 175.1	0.9276
	100	10			
	250	40			
	500	100			
	700	100			
Vincristine sulfate	0.25	30	0.68	Y = 9.885∗X + 43.32	0.7290
	0.50	50			
	1	70			
	5	90			

MELS = Methanol Extract of ​*Lentinus squarrosulus*, ​MEDC^1^ = Methanol Extract of ​*Daldinia concentrica*, ​METL = Methanol Extract of ​*Trametes lactinea*, ​MEFC = Methanol Extract of ​*Fomitopsis cajanderi*, ​MEDC^2^ = Methanol Extract of ​*Daedaleopsis confragosa*.

### 4.4 Molecular docking analysis for the analgesic study

**Figure 3:**
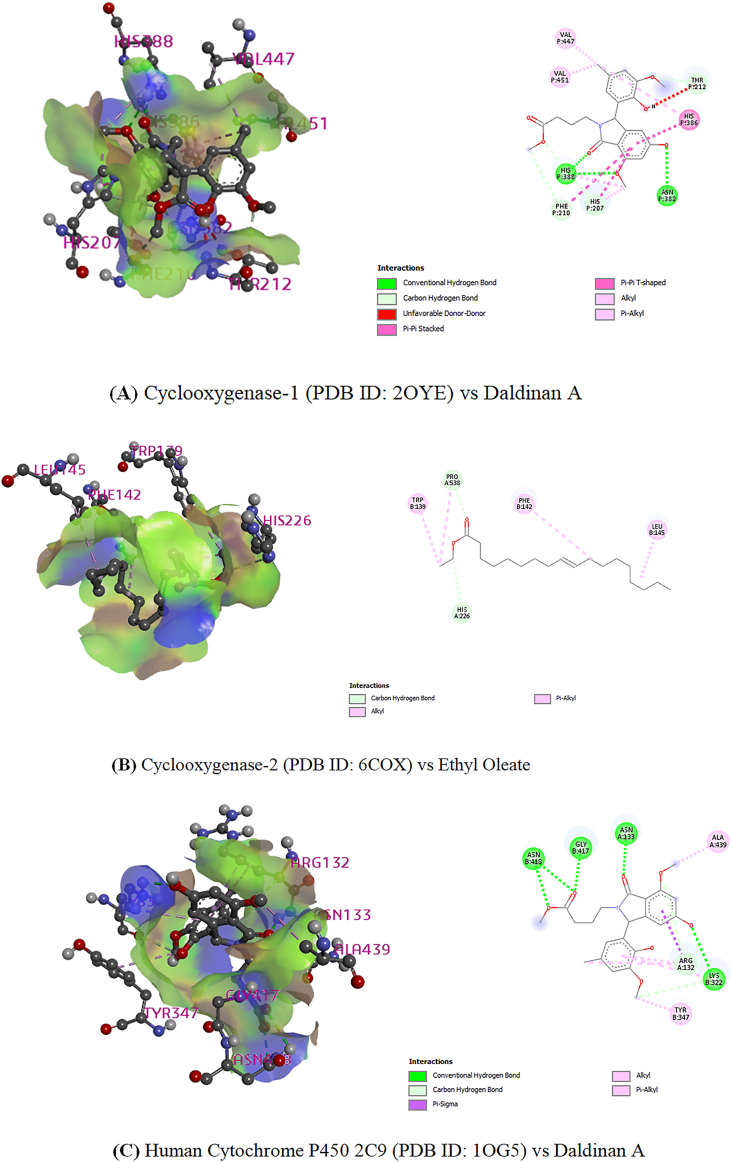
3D and 2D representation of vital interactions in the binding pocket of **(A)** Cyclooxygenase-1 (PDB ID: 2OYE) and Daldinan A, **(B)** Cyclooxygenase-2 (PDB ID: 6COX) and Ethyl oleate, and **(C)** Human Cytochrome P450 2C9 (PDB ID: 1OG5) with Daldinan A.

The docking analysis results for the analgesic activity have been presented in Table 2 and Figure 3. Completing the interaction of cyclooxygenase-1 (PDB ID: 2OYE) and the selected compounds, the highest score has been obtained -7.626 kcal/mol for daldinan A through the binding of his 207, phe 210, his 388, val 451, val 447, thr212, his 386 and asn 382 residue by a hydrogen bond. The ranking of the docking score is as follows: daldinan A > ethyl oleate > linoleic acid chloride > linoleic acid > oleic acid > palmitic acid. Besides, the ranking of the docking score of prostaglandin synthases-2 (PDB ID: 6COX) and selected components have been found as follow: Ethyl oleate > Linoleic acid chloride > Linoleic acid > Oleic Acid > Palmitic acid.

**Table 2:** Docking scores and glide energy of the selected compounds with the cyclooxygenase-1 (PDB ID: 2OYE), cyclooxygenase-2 (PDB ID: 6COX) for the analgesic and anti-inflammatory study, crystal structure of human cytochrome P450 2C9 (PDB ID: 1OG5).

Docking Score (kcal/mol)						
Compounds	Analgesic activity				Cytotoxic activity	
	Cyclooxygenase-1 (2OYE)		Cyclooxygenase-2 (6COX)		Cytochrome P450 2C9 (1OG5)	
	Docking score	Glide energy	Docking score	Glide energy	Docking score	Glide energy
Daldinan A	**-7.626**	-34.442	-	-	**-7.765**	-49.616
Ethyl oleate	-5.482	-38.732	**-5.396**	-27.672	-5.391	-39.285
Fomlactone A	-	-	-	-	-7.638	-46.39
Fomlactone B	-	-	-	-	-6.434	-46.87
Linoleic acid	-3.065	-38.405	-2.245	-27.943	-3.125	-35.827
Linoleic acid chloride	-4.007	-45.106	-3.490	-20.978	-2.889	-35.784
Oleic Acid	-1.928	-32.931	-1.735	-28.057	-2.676	-34.184
Palmitic acid	-1.435	-30.059	+0.420	-18.719	-0.52	-29.312

The bold data defines the best binding affinity to the receptors.

### 4.5 Molecular docking analysis for the cytotoxic study

From the results of molecular docking of cytotoxic study, all the nominated compounds were found to be interacting with the crystal structure of human cytochrome P450 2C9 (PDB ID: 1OG5). Daldinan A was estimated to score the highest docking score after completing the interaction with the crystal structure of human cytochrome P450 2C9 by bonding with the hydrogen bond through amino acid residues of human cytochrome P450 2C9 named asn 418, gly 417, asn 133, ala 439, lys 322, arg 132 and tyr347. The ranking of the docking score is as follow: daldinan A > fomlactone A > fomlactone B > ethyl oleate > linoleic acid > linoleic acid chloride > oleic acid > palmitic acid (Table 2 and Figure 3).

### 4.6 ADME/T analysis

Daldinan A; ethyl oleate; fomlactone A; fomlactone B; linoleic acid; linoleic acid chloride; oleic acid; palmitic acid meet the requirement of Lipinski's rules which might be measured as drug-likeliness (Table 3). All other compounds violated no more than one rule except fomlactone A that violated two rules (molecular weight and high lipophilicity).

**Table 3:** Absorption, digestion, metabolism, excretion, and toxicological (ADME/T) properties of the compounds for good oral bioavailability.

Molecules	PID	MW (g/mol)	HBD	HBA	LogP (o/w)	HIA	AM	CAR (binary)	PPB (100%)	AOT (kg/moL)
Daldinan A	56954714	415.4	2	7	2.92	0.9898	0.6700	0.9000	0.981	2.81
Ethyl oleate	5363269	310.5	0	2	6.59	0.9567	0.9000	0.5714	0.897	1.742
Fomlactone A	11071470	526.7	0	5	7.23	0.9538	0.7200	0.9429	0.965	2.947
Fomlactone B	11826996	484.72	1	4	6.66	0.9524	0.7400	0.9000	1.025	3.281
Linoleic acid	5280450	280.4	1	1	5.88	0.9087	0.9700	0.6714	0.921	1.605
Linoleic acid chloride	9817754	298.9	1	0	6.57	0.9236	0.5400	0.6000	1.018	1.895
Oleic Acid	445639	282.5	1	1	6.11	0.9087	0.9900	0.6714	0.886	1.696
Palmitic acid	985	256.42	1	1	5.55	0.8417	1.0000	0.6571	0.851	1.376

PID = PubChem ID, MW = Molecular Weight (acceptance range: <500), HBD = Hydrogen Bond Donor (acceptance range: ≤ 5), HBA = Hydrogen Bond Acceptor: (acceptance range: ≤ 10), LogP = High Lipophilicity (acceptance range: <5), HIA = Human Intestinal Absorption, AM = AMES Mutagenesis, CAR = Carcinogens, PPB = Plasma Protein Binding, AOT = Acute Oral Toxicity.

## 5 Discussion

The easiest and precise method for determining the peripheral analgesic method is the acetic acid-induced method (Uddin et al., 2018). When acetic acid is injected intraperitoneally, the level of inflammatory mediators like histamine, serotonin, bradykinin, cytokines, prostaglandins and leukotriene is detected in an increased amount in the fluid of peripheral tissue. After entering into the dorsal horn of CNS, these inflammatory mediators stimulate the primary afferent nociception. As a result, pain and writhing mediate to the acetic acid-induced mice (Islam et al., 2016). The less writhing count, the more analgesic activity of the test samples (Alam et al., 2020). The number of writhing declined as the doses were increased from 200 mg/kg to 400 mg/kg of body weight, demonstrating that the samples' behavior is dose-dependent. Compare to the control group; all the fungi extract significantly (*p* < 0.001) reduced the number of writhing in mice compared to the control. These fungi extracts may exert analgesic action by suppressing the synthesis or release of the inflammatory mediators. Methanol extract of *L. squarrosulus* (400 mg/kg) yielded 57.95% of the inhibition. Besides, *D. concentrica*, *T. lactinea*, *F. cajanderi* and *D. confragosa* (400 mg/kg, b.w; p.o) attained 63.26%, 67.80%, 62.12% and 65.53% of the pain inhibition subsequently. The maximum writhing inhibition has been possessed for the diclofenac sodium and METL 400 mg/kg. METL 400 mg/kg retarded the pain with the significant inhibition of writhing. After the administration of METL 400 mg/kg, only 17.0 ± 1.18 writhing were observed till the fixed time. The result possesses strong analgesic activities of the extracts. The fungi have been reported to possess analgesic activity owing to the presence of bio-constituents like daldinan A, ethyl oleate, fomlactone A–B, palmitic acid, linoleic acid, linoleic acid chloride, and oleic acid in the extracts (Mota et al., 2015). Brine shrimp nauplii lethality assay is one of the most simple methods for assessing the cytotoxicity of the crude extracts (Karchesy et al., 2016). This protocol is cost-effective and requires a small number of test materials (Bastos et al., 2009). It is a simple method of determining the anticancer, fungicidal and insecticidal action of the extracts (Karchesy et al., 2016). According to the study, extracts with the LC_50_ value greater than 1000 μg/mL are recommended as non-toxic (Déciga-Campos et al., 2007). The other criteria for extracts LC_50_ value: ≥ 500 ≤ 1000 μg/mL (weak toxicity), ≥100 ≤ 500 μg/mL (moderate toxicity) and <100 μg/mL (strong toxicity) (M Nguta et al., 2012). All the mushroom extracts are considered strongly cytotoxic as their LC_50_ value is less than 100 μg/mL (Table 1). The rate of the brine shrimp nauplii lethality was found to be increased with the rising concentration of the test samples. The fungi contain fatty acids like daldinan A, ethyl oleate, fomlactone A–B, palmitic acid, linoleic acid, linoleic acid chloride, and oleic acid and the other fatty acids were reported to having cytotoxic activity (Jóźwiak et al., 2020). The extracts can be a possible source of cytotoxic substances because of the occurrence of many functional phytochemicals such as fatty acids, polyphenols, flavonoids, saponins, steroids, and alkaloids in fungal extracts and it has been reported that some fatty acid and polyphenols destruct the membrane structure (Hossain et al., 2002, 2007; Jóźwiak et al., 2020). The exact mechanism of action for generating analgesic and cytotoxicity is not known. However, bioactive constituents like fatty acids may exert the analgesic and cytotoxic effects of the fungi. The bioassay results frequently correlate with more different bioactivity experiments (Karchesy et al., 2016). Nowadays, to predict the affinity of drug-target binding affinity and better understand the probable molecular mechanism of the therapeutic responses computational approach is considered (Emon et al., 2021a). Consequently, *in silico* molecular docking evaluations between ligands and protein were conducted to establish the clarity between mechanisms and their findings with the experimental results. Studies on molecular docking have been widely used to forecast ligand-target interactions and gain a deeper understanding of natural products' biological activity (Emon et al., 2020). Ligand–protein interaction and natural product's biological activity have been understood easily by molecular docking approaches. It also provides basic knowledge about the interaction and possible mechanisms of different protein binding sites (Khan et al., 2019). To get a clear insight into the biological behavior of the five wild mushrooms, eight isolated compounds were chosen for docking studies. These compounds are docked with three receptors, namely cyclooxygenase-1 (PDB ID: 2OYE) and prostaglandin synthases 2 (PDB ID: 6COX) for the analgesic study and crystal structure of human cytochrome P450 2C9 (PDB ID: 1OG5) that demonstrated greater binding affinity to the compounds. The lower molecular weight, lipophilicity and hydrogen bond capacity of these compounds could be shown to be highly permeable, good absorptive and bioavailable (Duffy et al., 2015; Lipinski et al., 1997). Based on this theory, daldinan A, ethyl oleate, fomlactone A, fomlactone B, lnoleic acid, linoleic acid chloride, oleic acid, and palmitic acid obey the laws of the Lipinski and thus show drug-like features.

## 6 Conclusion

Methanol extract of chosen wild mushrooms have been studied for *in vivo* analgesic and *in vitro* cytotoxic activities, whereas mainly known metabolites have been examined *in silico* approach. The overall results of this experiment showed significant analgesic and cytotoxic activity of the mushroom extracts. The study also suggested the drug-likeness of the metabolites from the selected mushrooms. However, it is essential to investigate the mushrooms again so that the fundamental mechanisms of the analgesic and cytotoxic activities of the mushroom extracts can be understood.

## Declarations

### Author contribution statement

S.M. Moazzem Hossen: Conceived and designed the experiments; Contributed reagents, materials, analysis tools or data; Wrote the paper.

Mohammad Shahadat Hossain, Nazim Uddin Emon: Conceived and designed the experiments; Performed the experiments; Analyzed and interpreted the data; Wrote the paper.

Sumaiya Akbar, Umme Tahmida: Performed the experiments; Analyzed and interpreted the data.

Jannatul Mawa: Analyzed and interpreted the data.

### Funding statement

This research did not receive any specific grant from funding agencies in the public, commercial, or not-for-profit sectors.

### Data availability statement

Data included in article/supplementary material/referenced in article.

### Declaration of interests statement

The authors declare no conflict of interest.

### Additional information

No additional information is available for this paper.
